# International Study to Evaluate PCR Methods for Detection of *Trypanosoma cruzi* DNA in Blood Samples from Chagas Disease Patients

**DOI:** 10.1371/journal.pntd.0000931

**Published:** 2011-01-11

**Authors:** Alejandro G. Schijman, Margarita Bisio, Liliana Orellana, Mariela Sued, Tomás Duffy, Ana M. Mejia Jaramillo, Carolina Cura, Frederic Auter, Vincent Veron, Yvonne Qvarnstrom, Stijn Deborggraeve, Gisely Hijar, Inés Zulantay, Raúl Horacio Lucero, Elsa Velazquez, Tatiana Tellez, Zunilda Sanchez Leon, Lucia Galvão, Debbie Nolder, María Monje Rumi, José E. Levi, Juan D. Ramirez, Pilar Zorrilla, María Flores, Maria I. Jercic, Gladys Crisante, Néstor Añez, Ana M. De Castro, Clara I. Gonzalez, Karla Acosta Viana, Pedro Yachelini, Faustino Torrico, Carlos Robello, Patricio Diosque, Omar Triana Chavez, Christine Aznar, Graciela Russomando, Philippe Büscher, Azzedine Assal, Felipe Guhl, Sergio Sosa Estani, Alexandre DaSilva, Constança Britto, Alejandro Luquetti, Janis Ladzins

**Affiliations:** 1 Laboratorio de Biología Molecular de la Enfermedad de Chagas (LabMECh), Instituto de Investigaciones en Ingeniería Genética y Biología Molecular (INGEBI-CONICET), Buenos Aires, Argentina; 2 Instituto de Cálculo, Universidad de Buenos Aires (UBA), Buenos Aires, Argentina; 3 Grupo Chagas, Universidad de Antioquia, Medellín, Colombia; 4 French Blood Services, La Plaine Saint Denis, Paris, France; 5 Laboratorio Hospitalario, Universidad de Parasitología, Cayene, French Guiana; 6 Department of Parasitic Diseases, Centers for Disease Control, Atlanta, Georgia, United States of America; 7 Institute of Tropical Medicine, Antwerp, Belgium; 8 Instituto Nacional de Salud, Lima, Perú; 9 Facultad de Medicina, Santiago de Chile, Chile; 10 Universidad Nacional del Nordeste, Chaco, Argentina; 11 Instituto Nacional de Chagas, Fatala Chabén, Buenos Aires, Argentina; 12 Centro Universitario de Medicina Tropical, Facultad de Medicina, Universidad Mayor de San Simon, Cochabamba, Bolivia; 13 Instituto de Investigaciones en Ciencias de la Salud, Universidad Nacional de Asunción, Asunción del Paraguay, Paraguay; 14 Faculdade de Farmácia, Petrópolis, Natal, Rio Grande do Norte, Brazil; 15 London School of Tropical Medicine and Hygiene Department of Clinical Parasitology, Hospital for Tropical Diseases, London, United Kingdom; 16 Laboratorio de Patología Experimental, Universidad Nacional de Salta, Salta, Argentina; 17 Blood Bank, Hospital Sirio Libanês, São Paulo, Brazil; 18 Centro de Investigaciones en Microbiología y Parasitología Tropical, Universidad de los Andes, Bogotá, Colombia; 19 Instituto Pasteur, Montevideo, Uruguay; 20 Centro de Mahahonda, Centro Nacional de Microbiologia, Instituto de Salud Carlos III, Madrid, España; 21 Sección Parasitología, Instituto Nacional De Salud, Santiago de Chile, Chile; 22 Centro de Investigaciones Parasitológicas “J.F. Torrealba,” Universidad de los Andes, Mérida, Venezuela; 23 Instituto de Patologia Tropical e Saúde Pública (IPTSP), Universidade Federal de Goiás, Goiânia, Brazil; 24 Grupo de Inmunología y Epidemiología Molecular (GIEM), Facultad de Salud, Universidad Industrial de Santander, Bucaramanga, Colombia; 25 Departamento de Biomedicina de Enfermedades Infecciosas y Parasitarias Laboratorio de Biología Celular, Centro de Investigaciones Regionales (CIR) “Dr Hideyo Noguchi,” Universidad Autónoma de Yucatán, Yucatán, México; 26 Instituto de Biomedicina, Universidad Católica de Santiago del Estero, Santiago del Estero, Argentina; 27 Centro Nacional de Diagnóstico e Investigación de Endemoepidemias (CeNDIE) ANLIS Dr. Carlos G. Malbrán, Buenos Aires, Argentina; 28 Laboratório de Biologia Molecular e Doenças Endêmicas, Instituto Oswaldo Cruz/FIOCRUZ, Rio de Janeiro, Brazil; 29 Laboratório de Pesquisa de Doença de Chagas, Goiãnia, Brazil; 30 Special Programme for Research and Training in Tropical Diseases (TDR), World Health Organization (WHO), Geneve, Switzerland; New York University School of Medicine, United States of America

## Abstract

**Background:**

A century after its discovery, Chagas disease still represents a major neglected tropical threat. Accurate diagnostics tools as well as surrogate markers of parasitological response to treatment are research priorities in the field. The purpose of this study was to evaluate the performance of PCR methods in detection of *Trypanosoma cruzi* DNA by an external quality evaluation.

**Methodology/Findings:**

An international collaborative study was launched by expert PCR laboratories from 16 countries. Currently used strategies were challenged against serial dilutions of purified DNA from stocks representing *T. cruzi* discrete typing units (DTU) I, IV and VI (set A), human blood spiked with parasite cells (set B) and Guanidine Hidrochloride-EDTA blood samples from 32 seropositive and 10 seronegative patients from Southern Cone countries (set C). Forty eight PCR tests were reported for set A and 44 for sets B and C; 28 targeted minicircle DNA (kDNA), 13 satellite DNA (Sat-DNA) and the remainder low copy number sequences. In set A, commercial master mixes and Sat-DNA Real Time PCR showed better specificity, but kDNA-PCR was more sensitive to detect DTU I DNA. In set B, commercial DNA extraction kits presented better specificity than solvent extraction protocols. Sat-DNA PCR tests had higher specificity, with sensitivities of 0.05–0.5 parasites/mL whereas specific kDNA tests detected 5.10^−3^ par/mL. Sixteen specific and coherent methods had a Good Performance in both sets A and B (10 fg/µl of DNA from all stocks, 5 par/mL spiked blood). The median values of sensitivities, specificities and accuracies obtained in testing the Set C samples with the 16 tests determined to be good performing by analyzing Sets A and B samples varied considerably. Out of them, four methods depicted the best performing parameters in all three sets of samples, detecting at least 10 fg/µl for each DNA stock, 0.5 par/mL and a sensitivity between 83.3–94.4%, specificity of 85–95%, accuracy of 86.8–89.5% and kappa index of 0.7–0.8 compared to consensus PCR reports of the 16 good performing tests and 63–69%, 100%, 71.4–76.2% and 0.4–0.5, respectively compared to serodiagnosis. Method LbD2 used solvent extraction followed by Sybr-Green based Real time PCR targeted to Sat-DNA; method LbD3 used solvent DNA extraction followed by conventional PCR targeted to Sat-DNA. The third method (LbF1) used glass fiber column based DNA extraction followed by TaqMan Real Time PCR targeted to Sat-DNA (cruzi 1/cruzi 2 and cruzi 3 TaqMan probe) and the fourth method (LbQ) used solvent DNA extraction followed by conventional hot-start PCR targeted to kDNA (primer pairs 121/122). These four methods were further evaluated at the coordinating laboratory in a subset of human blood samples, confirming the performance obtained by the participating laboratories.

**Conclusion/Significance:**

This study represents a first crucial step towards international validation of PCR procedures for detection of *T. cruzi* in human blood samples.

## Author Summary

A century after its discovery, Chagas disease, caused by the parasite *Trypanosoma cruzi*, still represents a major neglected tropical threat. Accurate diagnostics tools as well as surrogate markers of parasitological response to treatment are research priorities in the field. The polymerase chain reaction (PCR) has been proposed as a sensitive laboratory tool for detection of *T. cruzi* infection and monitoring of parasitological treatment outcome. However, high variation in accuracy and lack of international quality controls has precluded reliable applications in the clinical practice and comparisons of data among cohorts and geographical regions. In an effort towards harmonization of PCR strategies, 26 expert laboratories from 16 countries evaluated their current PCR procedures against sets of control samples, composed by serial dilutions of *T.cruzi* DNA from culture stocks belonging to different lineages, human blood spiked with parasite cells and blood samples from Chagas disease patients. A high variability in sensitivities and specificities was found among the 48 reported PCR tests. Out of them, four tests with best performance were selected and further evaluated. This study represents a crucial first step towards device of a standardized operative procedure for *T. cruzi* PCR.

## Introduction

A century after its discovery [1] Chagas disease still represents a health threat to an estimated 28 million people in the Americas, being the second highest illness burden among neglected tropical diseases [2]–[3]. The infection by the protozoan *Trypanosoma cruzi* can be acquired from blood-sucking triatomine bugs, blood transfusion, transplacental transmission or by the oral contamination foodstuffs by infected triatomine faeces [2]–[3].

Since 1990, a series of international initiatives based on vector control, systematic screening of blood donors in all endemic countries, and detection and treatment of congenital transmission have been launched for control and elimination of Chagas disease. These strategies have led to significant reduction in the number of infected people worldwide. According to information from 21 countries where the disease is endemic, the number of infected persons today is estimated to be 7,694,500, most of them at the chronic stage of disease [2]–[3].

Traditional parasitological procedures, such as xenodiagnosis and haemoculture are laborious and time-consuming and show poor sensitivities in cases of low-level parasitaemias, limiting their usefulness in diagnosis and monitoring of drug efficacy [4]–[6]. Since the past decade, the application of polymerase chain reaction (PCR) to detect *T. cruzi* directly in blood samples has opened new possibilities for the diagnosis of infection and evaluation of trypanocidal chemotherapy in different clinical and epidemiological settings [7]–[22]. These PCR procedures have revealed highly variable levels of sensitivity and specificity, depending on a number of technical factors such as, the volume of sample collected, the conditions of conservation of the sample, the methods used to isolate DNA, the parasite sequences and primers selected, the reagents used as well as the thermo-cycling conditions. Variability in PCR sensitivity could also be in part explained by the intermittent presence and quantity of circulating parasites at the time of blood collection. In addition, molecular targets from strains belonging to six different *T. cruzi* discrete typing units (DTUs, [23]) with dissimilar DNA content and gene dosage [22], [24]–[25] have been used for molecular diagnosis by different laboratories. In addition, sequence polymorphisms within amplified fragments among strains from different DTUs may influence the efficiency of amplification [26]–[27]. Moreover, false negative findings due to interference of PCR inhibitory substances co-purified during lysis and DNA extraction of blood samples and false positive results mostly due to carry over DNA contamination [22], [28] may arise.

In this context, the assessment of the performances of currently available PCR tests for detection of *T. cruzi* infection in blood samples and DNA control sets was launched by expert laboratories in PCR detection of *T. cruzi* infection from different countries of America and Europe. We aimed to compare the performance of currently used PCR strategies for detection of *T. cruzi* DNA in sets of blind samples, including purified DNA from reference culture stocks from different *T. cruzi* discrete typing units, human blood samples spiked with cultured parasite cells and clinical samples from seropositive and seronegative patients from different endemic countries, in order to select the best performing tests for validation.

## Materials and Methods

The participating laboratories were selected on the basis of their expertise in current processing of clinical samples for PCR detection of *T. cruzi* DNA, facilities with pre-PCR and post-PCR working areas and sufficient financial support to enable sustainability of PCR work after conclusion of this study. Twenty six Laboratories from different countries of America and Europe participated in this study, one laboratory from Belgium, Bolivia, France, French Guiana, Mexico, Paraguay, Peru, Spain, United Kingdom, Uruguay, USA, Venezuela, two laboratories from Chile, three from Colombia, four from Argentina and five from Brazil.

Aiming to explore the highest extent of currently used PCR tests for detection of *T. cruzi* DNA, the participating laboratories were encouraged to carry out all available PCR strategies currently in use according to their own protocols and using their own financial resources (LbX1 to LbXN in Table 1, where LbX denotes laboratory and the number denotes a given test).

**Table 1:** PCR tests reported by the participating Laboratories.

Lb/Test	Extraction Method	Target	Primer Names	PCR	Master Mix	Cycles
LbA	Solvent extraction	kDNA	121-122	Conventional	In-House	35
LbB	Solvent extraction	kDNA	S35 - S36	Conventional	In-House	30
LbC/1	Solvent extraction	kDNA	S35 - S36	Conventional	In-House	32
LbC/2	Solvent extraction	Sat-DNA	tcz1 - tcz2	Conventional	In-House	40
LbC/3	Solvent extraction	24s	D71-D71	Conventional	In-House	40
LbC/4	Solvent extraction	CO II-DNA	Tcmit 31-40	Conventional	In-House	48
Lb/C5	Solvent extraction	CO II-DNA	Nested Tcmit 10-21	Conventional	In-House	48
Lb/C6	Solvent extraction	SL-DNA	Tcc- Tc1-Tc2	Conventional	In-House	30
Lb/D1	Solvent extraction	kDNA	121-122	Conventional	In-House	36
Lb/D2	Solvent extraction	Sat-DNA	TczF-TczR	Real Time	Qiuantitect (Kt)	50
Lb/D3	Solvent extraction	Sat-DNA	TczF-TczR	Conventional	In-House	41
LbE	Chelex Resine	kDNA	121-122	Conventional	In-House	35
LbF/1	Roche Silica gel col (Kt)	Sat-DNA	cruzi1-2	Real Time	Roche (Kt)	45
LbF/2	Roche Silica gel col (Kt)	kDNA	32f-148r	Real Time	Roche (Kt)	45
LbG/1	Qiagen DNeasy Tissue kit (Kt)	kDNA FAM – IPC	32f-148r	Real Time	Apllied Biosystem (Kt)	55
LbG/2	Qiagen DNeasy Tissue kit (Kt)	kDNA FAM	32f-148r	Real Time	Apllied Biosystem (Kt)	55
LbG/3	Qiagen DNeasy Tissue kit (Kt)	kDNA VIC	32f-148r	Real Time	Apllied Biosystem (Kt)	55
LbG/4	Qiagen DNeasy Tissue kit (Kt)	Sat-DNA	cruzi1-2	Real Time	Apllied Biosystem (Kt)	45
LbH/1	Favorgen Glass fibers col (Kt)	kDNA	121-122	Conventional	GoTaq (Kt)	33
LbH/2	Favorgen Glass fibers col (Kt)	kDNA	121-122	Conventional	In-House	33
LbI/1	Favorgen Glass fibers col (Kt)	kDNA	121-122	Conventional	In-House	40
LbI/2	Favorgen Glass fibers col (Kt)	kDNA	S35 - S36	Conventional	In-House	40
LbJ	Solvent extraction	Sat-DNA	Tcz1-Tcz2	Conventional	In-House	40
LbK/1	Silica gel col (Kt)	Sat-DNA	cruzi1-2	Real Time	In-House	40
LbK/2	Silica gel col (Kt)	kDNA	121-122	Conventional	In-House	40
LbL/1	Blood mini Kit (Kt)	Sat-DNA	cruzi1-2	Conventional	In-House	40
LbL/2	Blood mini Kit (Kt)	Sat-DNA	Satellite DNA based kit	Conventional	OligoC-T Coris (Kt)	40
LbM	Silica gel col (Kt)	kDNA	TC1-TC2	Conventional	In-House	40
LbN/1	Solvent extraction	kDNA	121-122	Conventional	In-House	40
LbN/2	Solvent extraction	Sat-DNA	Tcz1-Tcz2	Conventional	In-House	35
LbO	Solvent extraction	kDNA	121-122	Conventional	In-House	40
LbP/1	Solvent extraction	kDNA	121-122	Conventional	In-House	35
LbP/2	CTAB (IH)	kDNA	121-122	Conventional	In-House	35
LbQ	Solvent extraction	kDNA	121-122	Conventional	In-House	37
LbR	Roche Silica gel col (Kt)	kDNA	121-122	Conventional	In-House	40
LbS/1	Qiagen Silica gel col (Kt)	18s	Tc18s F3-R4	Conventional	AmpliTaq Gold (Kt)	40
LbS/2	Qiagen Silica gel col (Kt)	Sat-DNA	cruzi1-2	Real Time	Platinum qPCR (Kt)	40
LbS/3	Qiagen Silica gel col (Kt)	18s	Tc18s F1042- R1144	Real Time	Platinum qPCR (Kt)	40
LbS/4	Qiagen Silica gel col (Kt)	kDNA	121-122	Conventional	AmpliTaq Gold (Kt)	40
LbT	ATGEN kit (Kt)	kDNA	121-122	Real Time	Invitrogen (Kt)	40
LbU/1	Solvent extraction	kDNA	121-122	Conventional	In-House	40
LbU/2	Solvent extraction	24s	D71-D72	Conventional	In-House	32
LbV/1	Silica gel col (Kt)	kDNA	121-122	Conventional	In-House	40
LbV/2	Silica gel col (Kt)	Sat-DNA	Tcz1-Tcz2	Conventional	In-House	30
LbW	Solvent extraction	kDNA	121-122	Conventional	In-House	40
LbX	Solvent extraction	kDNA	121-122	Conventional	In-House	35
LbY	Solvent extraction	kDNA	121-122	Conventional	In-House	35
LbZ	Silica gel col (Kt)	Sat-DNA	cruzi1-2	Real Time	TaqMan Univ (Kt)	45

LbX/1-6, Laboratory and test identification, kDNA, minicircle DNA; Sat-DNA, satellite DNA; 24s, 24sa rDNA; 18s, 18s rDNA; SL, Spliced Leader; kDNA FAM, kDNA TaqMan probe labeled with FAM; kDNA VIC, kDNA TaqMan probe labeled with VIC; IPC, TaqMan Exogenous Internal Positive Control (Applied Biosystems).

The organizing laboratory (LabMECh, INGEBI, Buenos Aires) was in charge of preparing characterised samples in three different sets (A, B and C), as described below.

Set A. This set consisted of ten-fold serial dilutions of *T. cruzi* DNA, plus three negative controls without DNA in bi-distilled sterile water. *T. cruzi* DNA was purified from epimastigote cells grown in LIT medium from stocks Silvio X10, Cl-Brener and CAN III, which are references for the discrete typing units *T. cruzi* I (DTU I), *T. cruzi* VI (DTU IIe) and *T. cruzi* IV (DTU IIa), respectively [23], [29]. The identity of the DTUs was confirmed using a PCR algorithm targeting several nuclear genes, as detailed in Burgos and coworkers [30]. *T. cruzi* DNA was extracted from parasite cultures using current chloroform- DNA extraction without vortexing during the procedure [31]. The concentration and quality of DNA was measured at 260/280 nm in triplicate, using a Nanodrop 1000 spectrophotometer (ThermoFisher Scientific, Waltham, MA, USA). Each series of DNA samples was conformed by concentrations ranging from 10 fg/ul to 10^−3^ fg/ul.

Set B. This set contained seronegative human blood samples treated with Guanidine Hidrochloride 6M-EDTA 0.2 M buffer, pH 8.00 [32] and spiked with ten-fold dilutions of cultured CL-Brener epimastigotes. One Guanidine Hidrochloride-EDTA treated blood sample without parasites was included as negative control. The samples were prepared as follows: a pool of human blood samples testing negative for *T. cruzi* infection in current serological methods and PCR was mixed with an equal volume of Guanidine Hidrochloride-EDTA buffer. An aliquot was withdrawn and spiked with 5 parasite cells/mL, homogenized, let stand overnight at room temperature, boiled the following day during 15 minutes [33], let stand at room temperature overnight and then stored at 4°C. The remaining non-spiked blood was treated in the same way and stored as a negative stock. One day later, the spiked Guanidine Hidrochloride-EDTA blood was used as starter for preparing 5 ten-fold serial dilutions, using as matrix the negative blood stock, to obtain samples ranging from 0.5 to 5.10^−5^ parasite equivalents/mL of blood.

Set C. This was a panel of 42 pre-characterized archived clinical blood samples stored in Guanidine Hidrochloride-EDTA buffer, including 10 from seronegative patients and 32 from seropositive patients from endemic regions of Argentina, Bolivia, Brazil and Paraguay. The seropositive panel was composed by patients at different phases of *T. cruzi* infection, namely, 2 immunosuppressed patients after heart transplantation, 23 indeterminate Chagas disease and 7 chronic Chagas disease patients with cardiac and/or digestive manifestations. They were selected from archived collections from the Serodiagnostic Laboratory for Chagas Disease, Federal University of Goias, Brazil, and from the organizing Lab. Diagnosis was based on their serological reactivity by at least two out of three routine serological methods (ELISA, IHA and Latex Agglutination tests) and clinical and electrocardiographic findings.

Samples from patients were obtained with written informed consent and approval of the Ethics Committee of the Rivadavia Hospital, Government of Buenos Aires city, Argentina and the Serodiagnostic Laboratory for Chagas Disease, Federal University of Goias, Goiania, Brazil. Furthermore, all samples were tested by two PCR tests performed on duplicate at the organizing laboratory, namely a hot-start PCR targeting kDNA according to Burgos et al [30], a Real time PCR targeting satellite DNA sequences and a Real time PCR targeting an internal amplification control, according to Duffy and coworkers [22], allowing confirmation of PCR negativity among seronegative samples and PCR positivity among a subgroup of the 32 seropositive samples (data not shown).

Each sample from set A, B and C was aliquoted and distributed into 1 ml Screw Top bar-coded tubes (Matrix Trackmates, UNITEK, USA) to each package. The packages were sent refrigerated to the participating laboratories (World Courier, Arg). Each laboratory received 50 µls of Set A and 500 µls of samples belonging to sets B and C.

### Best Performing PCR Methods

#### DNA extraction

Methods LbD2, LbD3 and LbQ: Solvent DNA extraction was carried out from 100 µl of Guanidine Hidrochloride-EDTA blood aliquots. Briefly, 100 µl aliquots were taken and well mixed with 100 µ l of phenol-chloroform-isoamylic alcohol (25∶24∶1) (phenol Tris–EDTA pH 8, USB Corporation, USA). After centrifugation for 3 min at 13000 rpm 150 µl of distilled water were added. The solution was mixed and centrifuged for 3 min at 13000 rpm. The aqueous phase was transferred to a clean tube, and a final extraction with 200 µ l of chloroform was performed. After centrifugation for 3 min at 13000 rpm the aqueous phase was transferred to a clean tube and mixed with 40 µg of rabbit liver glycogen (Sigma, USA). The DNA was precipitated with 200 µl of isopropyl alcohol during 30 minutes at −20°C. Then the solution was centrifuged at 13000 rpm for 15 min. The pellet was washed with 500 µl of 70% ethanol and centrifuged again 15 min at 13000 rpm. After discard the ethanol the pellet was allowed to dry during 10 min at 37°C. Finally the pellet was suspended in 50 µl 10 mM Tris-HCl, pH 8.5. DNA solution was stored at −20°C. Method LbF1: DNA isolation used a commercial kit (High Pure PCR Template preparation kit, Roche Applied Science) according to the manufacturer's protocol. DNA solution was stored at −20°C.

#### DNA amplification

Method LbD3 was carried out in a MJR PTC-100 thermocycler (MJ Research, Watertown, MA, USA). Master mix was composed by 1X Taq platinum amplification buffer, 250 µM deoxynucleotide triphosphate solution (dNTPs), 3 mM MgCl_2_ solution, 1,5 U Taq Platinum (Invitrogen, Brazil), 0.5 µM sat-DNA specific primers TCZ-F (GCTCTTGCCCACAMGGGTGC) and TCZ-R (CCAAGCAGCGGATAGTTCAGG), 5 µl of template DNA and a quantity of water sufficient to give a final volume of 50 µl. Cycling parameters were one step of 3 min at 94°C; 40 cycles of 45 sec at 94°C, 1 min at 68°C and 1 min at 72°C and one final extension step of 10 min at 72°C, 182 bp Sat-DNA PCR products were analysed in 3% agarose gels (Invitrogen, Life Technologies, USA) stained with ethidium bromide.

Method LbQ was carried out in a MJR PTC-100 thermocycler (MJ Research, Watertown, MA, USA). Master mix was composed by 1X Taq platinum amplification buffer, 200 µ M dNTPs, 3 mM MgCl_2_ solution, 1,5 U Taq Platinum (Invitrogen, Brazil), 10 µM kDNA specific primers 121 (AAATAATGTACGGGKGAGATGCATGA) and 122 (GGTTCGATTGGGGTTGGTGTAATATA), 7.5 µl of template DNA and a quantity of water sufficient to give a final volume of 50 µl. Cycling parameters were one step of 3 min denaturation at 94°C; 2 cycles of 1 min at 97.5°C, 2 min at 64°C; 33 cycles of 1 min at 94°C, 1 min at 62°C and one final extension step of 10 min at 72°C, 330 bp kDNA PCR products were analysed in 2% agarose gels stained with ethidium bromide.

Method LbD2 was conducted using a Rotor Gene 3000 (Corbett Research, Sydney, Australia) Real Time thermocycler. Each PCR reaction contained 1X Qiagen QuantiTect Sybr-Green PCR Master Mix (Qiagen), 0.5 µM SatDNA specific primers TCZ-F (GCTCTTGCCCACAMGGGTGC) and TCZ-R (CCAAGCAGCGGATAGTTCAGG), 2 µl of template DNA and PCR-grade H_2_O (Qiagen) to a final volume of 20 µl. The amplification was conducted under the following cycling conditions after 15 min of denaturation at 95°C, PCR amplification was carried out for 50 cycles (95°C for 10 s, 55°C for 15 s and 72°C for 10 s). Fluorescence data collection was performed at 72°C at the end of each cycle. After quantification, a melt curve was made with 74–85°C raising by 0.5°C each step and waiting for 4 seconds afterwards acquiring on Green channel. Melting temperture (Tm) of the amplicon was 81°C. Finally, data were analyzed with Rotor-Gene 6000 Series Software 1.7 (Corbett Research).

Method LbF1 was conducted using a Rotor Gene 3000 (Corbett Research, Sydney, Australia) Real Time thermocycler. Each PCR reaction contained 1X PCR FastStart Universal Probe Master Master Mix (Roche), 0.75 µM SatDNA specific primers cruzi 1 (ASTCGGCTGATCGTTTTCGA) and cruzi 2 (AATTCCTCCAAGCAGCGGATA), 0.25 µM SatDNA specific probe cruzi 3 (CACACACTGGACACCAA), 2 µl of template DNA and PCR-grade H_2_O to a final volume of 20 µl. The amplification was conducted under the following cycling conditions, after 15 min of denaturation at 95°C, 45 cycles at 95°C for 10 s, 54°C for 60 s. Fluorescence data collection was performed at 54°C at the end of each cycle. Finally, data were analyzed with Rotor-Gene 6000 Series Software 1.7 (Corbett Research).

The possibility of contamination of the PCR reagents and of the solutions used to prepare DNA was carefully examined through the use of appropriate controls. Also two dilutions from DNA purified from Cl- Brener strain were analyzed in each round as strong positive and detection limit control, respectively.

### Data Analysis

An access database form was distributed to the participants to standardize reporting of results. Those laboratories performing more than one PCR test per sample sent a separate report for each test. The results were analyzed by using SAS Software and Microsoft Excel. Due to the exploratory nature of the study, a descriptive analysis of results is provided.

For set A, the following parameters were evaluated: 1) specificity (Sp): the proportion of negative PCR results in the three negative samples, 2) coherence: (Co) the ability of reporting positive PCR findings in a consecutive way, from the highest to the lowest detected DNA concentration for each series of DNA dilutions of parasite stocks and 3) the detection limits (DL) for each stock. A test was defined as Good Performing Method (GPM) if it was 100% specific and coherent and capable of detecting 10 fg/ul or less DNA for all parasite DTU stocks.

For set B the same parameters were evaluated: Sp, Co and DL. A test was defined as GPM if it was 100% specific and coherent and capable of detecting 5 parasite equivalents/mL of Guanidine Hidrochloride-EDTA treated blood or less.

For each sample of set C, a consensus PCR result was obtained on the basis of the reports by GPM tests in sets A and B, as done in other PCR interlaboratory studies [34]. A sample was considered PCR positive by consensus if more than 50% of the GPM gave positive results and PCR negative if more than 50% of GPM tests gave negative results. Those samples for which 50% of the GPM methods gave positive reports and 50% gave negative ones were considered indeterminate.

The sensitivity, specificity, accuracy and kappa index of the different PCR tests were calculated by using 1) the above mentioned consensus PCR results and 2) the serological diagnosis as the reference methods.

Inter-observer kappa coefficients were calculated using GraphPad Software on-line statistical calculators (http://www.graphpad.com/quickcalcs/kappa1. cfm). Kappa values<0.01 indicate no concordance, those between 0.1 and 0.4 indicate weak concordance, those between 0.41 and 0.60 indicate clear concordance, those between 0.61 and 0.80 indicate strong concordance, and those between 0.81 and 1.00 indicate nearly complete concordance. Accuracy was calculated as reported [35].

## Results

Twenty six laboratories reported PCR results, using one to six different PCR tests (Table 1). The main sources of variability among laboratories and tests included DNA purification procedures using commercial kits (Kt) or in-house methods (IH), *T. cruzi* target and primer sequences, cycling instrumentation by conventional (C) or Real Time (RT) thermocyclers, cycling conditions, master mix compositions and trade marks of PCR kits (Kt) or reagents for IH master mixes.

A total of 48 PCR tests were reported for set A samples and 44 of them for sets B and C. Twenty eight tests targeted minicircle DNA, 24 of them amplified the 330 bp variable region and 4 amplified a 118 bp fragment from the constant region (Lb F2 and Lb G1 to G3, Table 1). Thirteen tests targeted the satellite DNA sequence (Sat-DNA), two targeted the 18s ribosomal RNA genes (18s rDNA), two amplified a fragment from the 24sα ribosomal RNA genes (24sα rDNA), one targeted the intergenic region of spliced-leader genes (SL-DNA) and two the mitochondrial gene for the subunit II of cytochrome oxidase (CO II-DNA) (Table 1).

### Analysis of Set A

Table 2 shows the data obtained by the 48 PCR tests on DNA dilutions from the 3 parasite stocks representing DTUs I, IV and VI. The seven PCR tests targeting sequences other than Sat-DNA or kDNA failed to detect the most concentrated DNA sample (10 fg/ul) of one, two or all three parasite stocks (Tests C3, C4, C6, S1 and S3; Detection Limit  =  ND), or reported false positive findings in the negative controls without DNA (Tests C5 and U2) and thus were not included in the following analysis.

**Table 2:** Performances of PCR tests in Sets A and B.

	Set A							Set B			GPM
		*T. cruzi* I		*T. cruzi* IV		*T. cruzi* VI		*T. cruzi* VI			
Lb/Test	Sp	Co	DL	Co	DL	Co	DL	Sp	Co	DL par/ml	
LbA	Y	Y	0.1	N	0.01	N	0.01	Y	N	0.005	N
LbB	Y	N	0.001	N	0.001	Y	0.1	Y	N	0.005	N
LbC/1	N	N	0.001	N	0.001	Y	0.1	N	Y	ND	N
LbC/2	Y	Y	ND	N	1	Y	10	**Y**	**Y**	**0.00005**	N
LbC/3	Y	Y	ND	Y	ND	Y	ND	NA	NA	NA	N
LbC/4	Y	Y	ND	Y	ND	Y	ND	NA	NA	NA	N
Lb/C5	N	Y	1	N	0.1	N	0.001	NA	NA	NA	N
Lb/C6	Y	Y	ND	Y	ND	Y	ND	NA	NA	NA	N
Lb/D1	**Y**	**Y**	**1**	**Y**	**10**	**Y**	**1**	N	N	0.005	N
**Lb/D2**	**Y**	**Y**	**1**	**Y**	**10**	**Y**	**1**	**Y**	**Y**	**0.05**	**Y**
**Lb/D3**	**Y**	**Y**	**1**	**Y**	**10**	**Y**	**1**	**Y**	**Y**	**0.05**	**Y**
**LbE**	**Y**	**Y**	**1**	**Y**	**10**	**Y**	**10**	**Y**	**Y**	**0.005**	**Y**
**LbF/1**	**Y**	**Y**	**0.1**	**Y**	**1**	**Y**	**0.01**	**Y**	**Y**	**0.05**	**Y**
LbF/2	Y	Y	1	N	0.01	Y	1	**Y**	**Y**	**0.5**	N
LbG/1	Y	Y	0.1	Y	1	Y	ND	**Y**	**Y**	**0.05**	N
**LbG/2**	**Y**	**Y**	**0.1**	**Y**	**1**	**Y**	**1**	**Y**	**Y**	**0.05**	**Y**
**LbG/3**	**Y**	**Y**	**0.1**	**Y**	**1**	**Y**	**1**	**Y**	**Y**	**0.05**	**Y**
**LbG/4**	**Y**	**Y**	**1**	**Y**	**1**	**Y**	**1**	**Y**	**Y**	**0.5**	**Y**
LbH/1	Y	Y	1	Y	10	N	0.001	Y	N	0.05	N
LbH/2	Y	Y	1	N	0.1	Y	10	Y	N	0.05	N
LbI/1	Y	Y	1	N	0.001	Y	10	**Y**	**Y**	**0.005**	N
**LbI/2**	**Y**	**Y**	**1**	**Y**	**10**	**Y**	**10**	**Y**	**Y**	**0.05**	**Y**
LbJ	Y	Y	0.01	N	0.001	N	0.001	Y	N	0.5	N
**LbK/1**	**Y**	**Y**	**10**	**Y**	**10**	**Y**	**10**	**Y**	**Y**	**0.5**	**Y**
**LbK/2**	**Y**	**Y**	**1**	**Y**	**10**	**Y**	**10**	**Y**	**Y**	**5**	N
LbL/1	Y	Y	ND	Y	10	Y	1	**Y**	**Y**	**0.5**	N
LbL/2	Y	Y	ND	Y	ND	Y	1	**Y**	**Y**	**0.5**	N
LbM	N	Y	0.001	Y	0.001	N	0.001	Y	Y	ND	N
LbN/1	Y	Y	0.1	Y	ND	N	0.1	Y	N	0.005	N
LbN/2	Y	Y	1	Y	ND	Y	10	**Y**	**Y**	**0.5**	N
LbO	Y	Y	10	N	1	Y	ND	**Y**	**Y**	**0.05**	N
**LbP/1**	**Y**	**Y**	**0.1**	**Y**	**10**	**Y**	**1**	**Y**	**Y**	**5**	N
**LbP/2**	**Y**	**Y**	**0.1**	**Y**	**10**	**Y**	**1**	**Y**	**Y**	**0.5**	N
**LbQ**	**Y**	**Y**	**1**	**Y**	**10**	**Y**	**1**	**Y**	**Y**	**0.5**	N
LbR	**Y**	**Y**	**0.1**	**Y**	**1**	**Y**	**0.1**	Y	N	0.00005	N
LbS/1	Y	Y	1	Y	ND	Y	ND	Y	Y	ND	N
**LbS/2**	**Y**	**Y**	**1**	**Y**	**10**	**Y**	**1**	**Y**	**Y**	**0.5**	N
LbS/3	Y	Y	10	Y	10	Y	ND	Y	Y	ND	N
LbS/4	**Y**	**Y**	**1**	**Y**	**1**	**Y**	**10**	N	N	0.00005	N
LbT	N	N	0.001	Y	1	N	0.01	N	N	0.05	N
LbU/1	N	Y	0.001	Y	0.001	Y	0.001	Y	N	0.005	N
LbU/2	N	Y	0.001	Y	0.001	Y	0.001	N	N	0.005	N
**LbV/1**	**Y**	**Y**	**0.1**	**Y**	**10**	**Y**	**10**	**Y**	**Y**	**0.05**	N
LbV/2	Y	Y	0.1	Y	ND	Y	10	**Y**	**Y**	**0.05**	N
**LbW**	**Y**	**Y**	**0.01**	**Y**	**1**	**Y**	**0.1**	**Y**	**Y**	**0.005**	N
LbX	N	N	0.01	N	0.001	N	0.1	Y	N	0.0005	N
LbY	N	Y	1	Y	1	Y	0.1	Y	Y	ND	N
LbZ	**Y**	**Y**	**1**	**Y**	**1**	**Y**	**0.01**	N	N	0.0005	N

LbX/1-6, Laboratory and test identification; Bold Type, Good Performing Methods in sets A or B; GPM, Good Performing Methods in sets A and B; Sp, 100% of specificity in all controls without *T. cruzi* DNA; Co, Coherence in PCR positive reports; DL, Detection limit in fg DNA/ul; Y, Affirmative; N, Negative; NA, Not available; ND, Not detectable**.**

**Figure 1:**
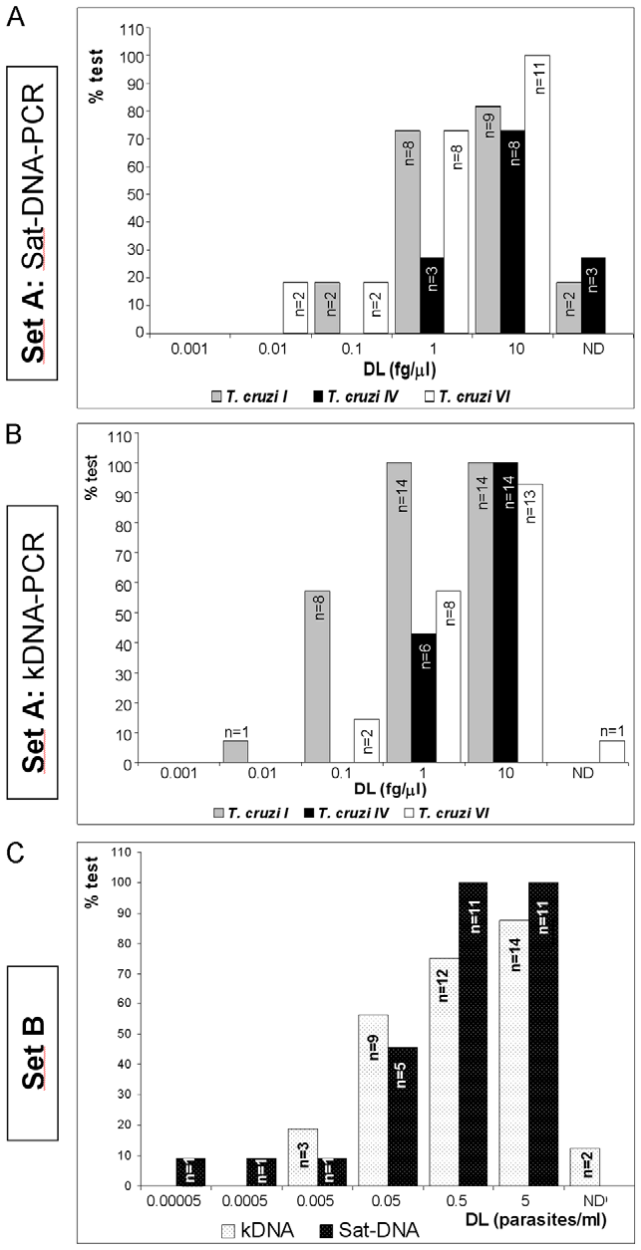
Analytical Sensitivity of specific and coherent PCR tests in sets A and B. Distribution of detection limits (DL) of specific and coherent PCR tests targeted to Sat-DNA (A) and kDNA sequences (B) for detecting serial dilutions of purified DNA from 3 parasite stocks (Set A) representative of *T. cruzi* DTU I (Silvio×10), DTU IV (Can III cl1) and DTU VI (Cl Brener). C. Distribution of detection limits (DL) of specific and coherent PCR tests targeted to Sat-DNA (black bars) and kDNA sequences (white bars) carried out from human blood spiked with serial dilutions of parasite cells (Set B).

Out of the 41 tests based on kDNA (28 tests) or Sat-DNA sequences (13 tests), 25 (51.2%) provided specific and coherent results for all three parasite stocks (Sp = Y, Co = Y, Table 2). Fourteen of them targeted kDNA, representing 50% of the reported kDNA-PCR tests and 11 targeted Sat-DNA, representing 84.6% of Sat-DNA PCR tests. These data indicated that PCR tests based on Sat-DNA sequences were more specific than those based on kDNA. Figures 1A and 1B show the distribution of the detection limits (DL) of the above mentioned 11 Sat-DNA PCR and 14 kDNA PCR tests, respectively, for each *T. cruzi* stock.

Analysis of *T. cruzi* I DNA series: Nine out of 11 Sat-DNA- and all 14 kDNA-PCR tests were capable of detecting at least the most concentrated *T. cruzi* I DNA sample (Figure 1 A and B, grey bars) and 2 Sat-DNA and 8 kDNA-PCR tests could detect 0.1 fg/µl of *T. cruzi* I DNA. The lowest detection limit for *T. cruzi* I DNA was 0.01 fg/ul obtained by laboratory W using conventional kDNA-PCR (Table 2 and Figure 1B). Thus, kDNA- PCR tests were more sensitive than Sat-DNA PCR tests to detect *T. cruzi* I DNA.

Analysis of *T. cruzi* IV DNA series: 8 out of 11 Sat-DNA- and all 14 kDNA-PCR tests were capable of detecting the most concentrated *T. cruzi* IV DNA sample (Figure 1 A and B, black bars). The lowest detection limit (1 fg/µl) was reached by three Sat-DNA- and six kDNA-PCR tests, suggesting similar analytical sensitivities of methods based on both molecular targets to detect *T. cruzi* IV DNA.

Analysis of *T. cruzi* VI DNA series: All 11 Sat-DNA- and 13 out of 14 kDNA-PCR tests were capable of detecting the most concentrated *T. cruzi* VI DNA sample (Figure 1 A and B, white bars). The only test that did not detect Cl-Brener DNA amplified the constant kDNA region (G1, kDNAc, Table 2). The lowest detection limit (0.01 fg/µl) was obtained by 2 Sat-DNA PCR tests (Z and F1, Table 2) followed by 0.1 fg/µl obtained by 2 conventional kDNA-PCR tests (R and W, Table 2).

Overall, the reported PCR tests were less sensitive for detecting DNA from the *T. cruzi* IV reference stock. Twenty PCR tests showing specific and coherent results and detecting at least the most concentrated DNA samples from each of the parasite stocks were considered Good Performing Methods for Set A (bold fonts, Table 2). They comprised 53.8% of 13 Sat-DNA-PCR and 46.42% of 28 kDNA-PCR tests. Ten GPM tests used in-house (IH) PCR mixtures and 10 used commercial master mixes (Kt), representing 35.7% of the 28 IH and 76.9% of the 13 Kt PCR reagent mixes. In addition, 12 GPM used conventional amplification and eight used real time PCR (C and RT in Table 2), representing 38.7% of 31 C and 72.7% of 11 RT tests. These data showed that commercial master mixes and real time PCR offered better PCR performance in purified DNA samples.

### Analysis of Set B

Out of the 44 PCR tests reported for spiked Guanidine Hidrochloride-EDTA blood samples, the three tests targeting sequences other than Sat-DNA or kDNA were not further analyzed, because they failed to detect the most concentrated sample (S1 and S3 tests) or showed false positive findings in the non-spiked control (U2 test) (Set B, Table 2).

Twenty five out of 41 PCR tests based on kDNA and Sat-DNA sequences showed specific, coherent results and detection limits of at least 5 par/ml (GPM, bold fonts, Table 2, Set B). They included 14 kDNA and 11 Sat-DNA PCR tests, representing 50% of 28 kDNA and 84.6% of 13 Sat-DNA based tests. Ten GPM used in-house extraction methods and 15 used DNA extraction kits, representing 41.6% of 24 IH and 62.5% of 24 Kt tests. Thus, methods using commercial DNA extraction and Sat-DNA as amplification target resulted in better performance. Procedures based on kDNA presented more variation in sensitivity than Sat-DNA tests (Figure 1C, white bars). The smallest detected concentration was 5×10^−3^ par/ml, recorded by three laboratories using conventional kDNA-PCR after DNA extractions with Chelex resine, a blood extraction kit or solvent extraction with Phenol (LbE, LbL1 and LbW, respectively, Table 1). Tests based on Sat-DNA presented sensitivities between 0.05 and 0.5 par/ml (10/11 tests, Figure 1C, black bars) with the only exception of one test based on solvent DNA extraction and IH conventional Sat-DNA PCR (C2, Table 1) that reached a detection limit of 5×10^−5^ par/mL (Table 2, and Figure 1C).

### Analysis of Set C

Out of the 44 PCR tests performed on clinical samples of Set C, a 18s-rDNA PCR (S3) and a SL-DNA PCR (C6) tests did not detect any positive sample and the 24s α rDNA-PCR test (U2) had only 40% of specificity. Consequently, they were not included for subsequent analysis. The levels of agreement among the 41 remaining PCR tests on the reports for each clinical sample are presented in Table 3. For each sample, 3 series of consensus PCR results were calculated: 1) consensus based on the 28 kDNA-PCR tests, 2) consensus based on the 13 Sat-DNA PCR tests and 3) consensus based on the 16 tests defined as GPM in both sets A and B samples. The sensitivity of consensus kDNA-PCR was 65.62% (21 PCR positive/32 seropositive samples), that of consensus Sat-DNA was 62.5% (20 PCR positive/32 seropositive samples) and that of consensus GPM was 56.25% (B (18 PCR positive/32 seropositive samples) being 4 samples indeterminate (13, 18, 20, 21, Table 3) because the levels of agreement among GPM tests was 50%.

**Table 3:** Concordance of PCR results reported for each clinical case of Set C.

CLINICAL CASES						kDNA PCR n = 28			Sat-DNA PCR n = 13			GPM n = 16		
ID	G	Ag	Status	Region	EN	pos/tot	%	Cons	pos/tot	%	Cons	pos/tot	%	Cons
1	F	NA	cChHD-HTx	Arg- Uk^1^	Uk	26	92,9	**P O S**	12	92,3	**P O S**	15	93,8	**P O S**
2	M	NA	cChHD-HTx	Arg-Chaco^1^	Yes	26	92,9	**P O S**	10	76,9	**P O S**	15	93,8	**P O S**
3	F	54	Mega III cChH	Br- MG	Yes	20	71,4	**P O S**	12	92,3	**P O S**	12	75,0	**P O S**
4	F	42	Pregnant	Arg- Salta^2^	Yes	18	64,3	**P O S**	12	92,3	**P O S**	14	87,5	**P O S**
5	F	25	Pregnant	Bo-Uk	Yes	18	64,3	**P O S**	11	84,6	**P O S**	12	75,0	**P O S**
6	M	20	Blood donor	Br-BA	Yes	19	67,9	**P O S**	10	76,9	**P O S**	10	62,5	**P O S**
7	F	41	cChHD	Br-BA	Yes	18	64,3	**P O S**	9	69,2	**P O S**	11	68,8	**P O S**
8	F	31	Pregnant	Bo-Uk	Yes	16	57,1	**P O S**	10	76,9	**P O S**	11	68,8	**P O S**
9	F	NA	Pregnant	Par-Uk2	Yes	15	53,6	**P O S**	11	84,6	**P O S**	12	75,0	**P O S**
10	F	22	Ex-pregnant	Br-BA	Yes	17	60,7	**P O S**	10	76,9	**P O S**	12	75,0	**P O S**
11	F	41	Chronic CD	Br-BA	Yes	16	57,1	**P O S**	9	69,2	**P O S**	11	68,8	**P O S**
12	F	24	Ex-pregnant	Br-Go	Yes	15	53,6	**P O S**	9	69,2	**P O S**	10	62,5	**P O S**
13	F	32	Pregnant	Arg-Co	Yes	16	57,1	**P O S**	8	61,5	**P O S**	8	50,0	**I N D**
14	F	35	Ex-pregnant	Br-Ceara	Yes	17	60,7	**P O S**	9	69,2	**P O S**	12	75,0	**P O S**
15	F	47	cChHD	Br-Go	Yes	17	60,7	**P O S**	8	61,5	**P O S**	9	56,3	**P O S**
16	F	NA	Pregnant	Par-Uk	Yes	14	50,0	**P O S**	8	61,5	**P O S**	9	56,3	**P O S**
17	M	55	CD	Br-MG	Yes	15	53,6	**P O S**	7	53,8	**P O S**	10	62,5	**P O S**
18	M	33	cChHD	Br-BA	Yes	15	53,6	**P O S**	7	53,8	**P O S**	8	50,0	**I N D**
19	F	66	Mega II + CBBB	Br-BA	Yes	15	53,6	**P O S**	5	38,5	N E G	9	56,3	**P O S**
20	F	18	Ex-pregnant	Br-Go	Yes	16	57,1	**P O S**	4	30,8	N E G	8	50,0	**I N D**
21	F	18	Pregnant	Arg-Sg	Yes	15	53,6	**P O S**	6	46,2	N E G	8	50,0	**I N D**
22	F	43	Indeterminate CD	Br-BA	Yes	13	46,4	N E G	7	53,8	**P O S**	6	37,5	N E G
23	F	57	Blood donor	Br-Piaui	Yes	11	39,3	N E G	8	61,5	**P O S**	9	56,3	**P O S**
24	F	46	Blood donor	Br-BA	Yes	7	25,0	N E G	6	46,2	N E G	6	37,5	N E G
25	F	25	Pregnant	Par-Uk^2^	Yes	10	35,7	N E G	5	38,5	N E G	5	31,3	N E G
26	F	32	Pregnant	Par-Uk^2^	Yes	11	39,3	N E G	5	38,5	N E G	6	37,5	N E G
27	F	36	Pregnant	Arg-Chaco	Yes	11	39,3	N E G	5	38,5	N E G	5	31,3	N E G
28	F	36	Pregnant	Arg-Chaco	Yes	9	32,1	N E G	4	30,8	N E G	2	12,5	N E G
29	M	59	cChHD	Br-Piaui	Yes	9	32,1	N E G	2	15,4	N E G	5	31,3	N E G
30	F	29	Mega-II	Br-Go	Yes	7	25,0	N E G	4	30,8	N E G	5	31,3	N E G
31	F	NA	Pregnant	Par-Uk^2^	Yes	9	32,1	N E G	1	7,7	N E G	1	6,3	N E G
32	F	28	Pregnant	Arg-Sg	Yes	2	7,1	N E G	3	23,1	N E G	1	6,3	N E G
33	M	38	Routine	Br-Go	No	4	14,3	N E G	1	7,7	N E G	1	6,3	N E G
34	M	51	Routine	Br-Uk	Yes	5	17,9	N E G	1	7,7	N E G	1	6,3	N E G
35	M	NA	Blood donor	Arg-BAs	No	6	21,4	N E G	1	7,7	N E G	1	6,3	N E G
36	F	39	Routine	Br-Go	Yes	8	28,6	N E G	1	7,7	N E G	1	6,3	N E G
37	F	37	Routine	Br-Go	No	6	21,4	N E G	1	7,7	N E G	2	12,5	N E G
38	M	NA	Blood donor	Arg-BA	No	7	25,0	N E G	2	15,4	N E G	3	18,8	N E G
39	F	36	Routine	Br-Bh	Yes	7	25,0	N E G	4	30,8	N E G	3	18,8	N E G
40	F	40	Routine	Br-Go	Yes	8	28,6	N E G	4	30,8	N E G	5	31,3	N E G
41	F	40	Routine	Br-Go	Yes	9	32,1	N E G	6	46,2	N E G	5	31,3	N E G
42	F	58	Routine	Br-Go	Yes	11	39,3	N E G	6	46,2	N E G	6	37,5	N E G

Patients 1 to 32 are seropositive and 33 to 42 seronegative. 28 kDNA tests and 13 Sat DNA tests were performed for each sample.

kDNA, minicircle DNA; Sat-DNA, satellite DNA; GPM Good performing Methods in panels A and B; ID, sample identification number; G, Gender; Ag, age in years; EN, Endemic precedence; %: Percentage of positive results; Cons, Consensus PCR result; F, female; M, male; NA, not available; 28 kDNA tests and 13 Sat DNA tests were performed for each sample.

1 *T.cruzi* DTU I,

2 *T.cruzi* DTU II/V/VI, NE, not endemic; Uk, Unknown; Pos, positive consensus; Ind, indeterminate consensus; Neg, negative consensus; cChHD, chronic Chagas heart disease, Mega Megacolon, CBBB, Complete Branch Bundle Blockage, HTx, Heart transplantation; Arg: Argentina; Bo: Bolivia; Br: Brazil; Par: Paraguay; BAs, Buenos Aires; Bh, Bahia; Go, Goias; MG: Minas Gerais; Sg: Santiago del Estero.

The individual performance of the 41 PCR tests was evaluated in comparison with the consensus PCR results reached by the 16 GPM in sets A and B (18 PCR positive, 20 PCR negative samples) and in comparison with serologic diagnosis (10 seronegative, 32 seropositive samples) (Table 4). There was a high variability among the performances of the different methods (Table 4). The median values of sensitivity, specificity and accuracy of the 41 tests were 72, 77.5 and 68.4%, respectively in comparison to consensus GPM PCR reports, and 59.4, 70 and 59.5%, respectively in comparison to serological diagnosis (Table 4).

**Table 4:** Performance of PCR tests in comparison to consensus GPM reports and serodiagnosis.

	Test		PCR performance vs consensus GPM K+S				PCR performance versus Serology				
Lb/Test	PCR	Target	Se	Sp	Acc	kappa	Se	Sp	Acc	kappa	BPM
			N = 18	N = 20	N = 38	N = 38	N = 32	N = 10	N = 42	N = 38	
LbA	C	K	33.3	60.0	47.4	−0.1	31	70	40.5	0.0	N
LbB	C	K	72.2	35.0	52.6	0.1	66	30	57.1	0.0	N
LbC/1	C	K	0.0	100.0	52.6	0.0	0	100	23.8	0.0	N
LbC/2	C	S	66.7	15.0	39.5	−0.2	69	10	54.8	−0.2	N
Lb/D1	C	K	94.4	45.0	68.4	0.4	81	40	71.4	0.2	N
**Lb/D2**	**RT**	**S**	**94.4**	**85.0**	**89.5**	**0.8**	**69**	**100**	**76.2**	**0.5**	Y
**Lb/D3**	**C**	**S**	**94.4**	**85.0**	**89.5**	**0.8**	**63**	**100**	**71.4**	**0.4**	Y
**LbE**	**C**	**K**	**94.4**	**65.0**	**78.9**	**0.6**	**81**	**80**	**81.0**	**0.5**	N
**LbF/1**	**RT**	**S**	**83.3**	**95.0**	**89.5**	**0.8**	**63**	**100**	**71.4**	**0.4**	Y
LbF/2	RT	K	72.2	90.0	81.6	0.6	53	90	61.9	0.3	N
LbG/1	RT	K	100.0	60.0	78.9	0.6	84	60	78.6	0.5	N
**LbG/2**	**RT**	**K**	**100.0**	**65.0**	**81.6**	**0.6**	**78**	**40**	**69.0**	**0.4**	N
**LbG/3**	**RT**	**K**	**100.0**	**65.0**	**81.6**	**0.6**	**78**	**40**	**69.0**	**0.4**	N
**LbG/4**	**RT**	**S**	**94.4**	**90.0**	**92.1**	**0.8**	**63**	**60**	**61.9**	**0.4**	N
LbH/1	C	K	27.8	80.0	55.3	0.1	22	80	35.7	0.0	N
LbH/2	C	K	22.2	80.0	52.6	0.0	16	80	31.0	0.0	N
LbI/1	C	K	83.3	40.0	60.5	0.2	78	50	71.4	0.3	N
**LbI/2**	**C**	**K**	**38.9**	**40.0**	**39.5**	**−0.2**	**53**	**40**	**50.0**	**−0.1**	N
LbJ	C	S	55.6	60.0	57.9	0.2	59	70	61.9	0.2	N
**LbK/1**	**RT**	**S**	**61.1**	**70.0**	**65.8**	**0.3**	**44**	**60**	**47.6**	**0.3**	N
**LbK/2**	**C**	**K**	**0.0**	**100.0**	**52.6**	**0.0**	**0**	**100**	**23.8**	**0.0**	N
LbL/1	C	S	88.9	45.0	65.8	0.3	84	60	78.6	0.4	N
LbL/2	C	S	83.3	60.0	71.1	0.4	72	60	69.0	0.3	N
LbM	C	K	66.7	50.0	57.9	0.2	59	50	57.1	0.1	N
LbN/1	C	K	66.7	80.0	73.7	0.5	47	60	50.0	0.0	N
LbN/2	C	S	72.2	80.0	76.3	0.5	47	70	52.4	0.1	N
LbO	C	K	66.7	55.0	60.5	0.2	47	30	42.9	−0.2	N
**LbP/1**	**C**	**K**	**88.9**	**85.0**	**86.8**	**0.7**	**53**	**80**	**59.5**	**0.2**	N
**LbP/2**	**C**	**K**	**11.1**	**100.0**	**57.9**	**0.1**	**6**	**100**	**28.6**	**0.0**	N
**LbQ**	**C**	**K**	**83.3**	**90.0**	**86.8**	**0.7**	**63**	**100**	**71.4**	**0.4**	Y
LbR	C	K	88.9	55.0	71.1	0.4	81	70	78.6	0.5	N
**LbS/2**	**RT**	**S**	**50.0**	**90.0**	**71.1**	**0.4**	**38**	**100**	**52.4**	**0.2**	N
LbS/4	C	K	55.6	90.0	73.7	0.5	47	100	59.5	0.3	N
LbT	RT	K	50.0	75.0	63.2	0.3	41	80	50.0	0.1	N
LbU/1	C	K	16.7	95.0	57.9	0.1	9	90	28.6	0.0	N
**LbV/1**	**C**	**K**	**27.8**	**100.0**	**65.8**	**0.3**	**16**	**100**	**35.7**	**0.1**	N
LbV/2	C	S	44.4	100.0	73.7	0.5	28	100	45.2	0.2	N
**LbW**	**C**	**K**	**100.0**	**35.0**	**65.8**	**0.3**	**91**	**40**	**78.6**	**0.3**	N
LbX	C	K	100.0	50.0	73.7	0.5	88	60	81.0	0.5	N
LbY	C	K	77.8	50.0	63.2	0.3	75	80	76.2	0.5	N
LbZ	RT	S	50.0	90.0	71.1	0.4	38	100	52.4	0.2	N
**Median**			**72**	**77.5**	**68.4**	**0.4**	**59.4**	**70.0**	**59.5**	**0.2**	
**(25-75p)**			**(50**–**88.9)**	**(55**–**90)**	**(57.9**–**781)**	**(0.2**–**0.6)**	**(37.5**–**75)**	**(60**–**100)**	**(47.6**–**71.4)**	**(0**–**0.4)**	

LbX/1-6, Laboratory and test identification; BPM, Best Performing Methods; Consensus GPM K + S: consensus findings of GPM by kDNA and Satellite DNA PCRs; C, Conventional PCR, RT, Real Time PCR; K, kDNA; S, Satellite DNA; Se, sensitivity; Sp, specificity; Acc, accuracy; kappa, kappa index; N, negative; Y, affirmative; 25–75p, 25th-75th percentiles; Bold type, Good Performing Methods (GPM) in sets A and B.

### Best Performing Methods

Four GPM showed the best operational parameters in set C (Table 4). Tests LbD2 and LbD3 used solvent DNA extraction followed by conventional hot-start and Real time PCR targeted to Sat-DNA, respectively (primer pairs TCZ-F/TCZ-R). Test LbF1 used a commercial kit for DNA extraction based on glass fiber columns and Real Time PCR targeted to Sat-DNA (primer pairs cruzi 1/cruzi 2 and TaqMan probe cruzi 3) and test LbQ used solvent DNA extraction and conventional hot-start PCR for kDNA (primer pairs 121/122).

**Figure 2:**
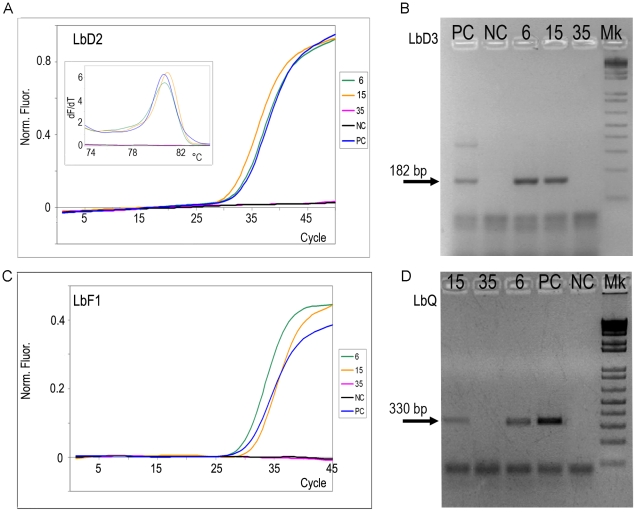
Examples of the outputs of the four best performing PCR methods. A. LbD2 ; B.LbD3, C. LbF1 and D. LbQ. The methods are described in Materials and Methods and Table 1. 6, 15: seropositive samples; 35; seronegative sample (Table 3). PC: Positive control: 10 fg/µl of T.cruzi VI. NC. Negative Control: Master Mixes devoid of DNA.

The performance of these four tests was further evaluated at the coordinating laboratory on a subset of samples from seropositive and seronegative patients, analysed in four independent experiments (Table 5). Examples of the outputs of each method are shown in Figure 2. The degree of concordance among the reported results by the BPM was between 87.5% and 90.62%. This intralaboratory evaluation showed that the selected methods depicted similar operational parameters than when performed by the corresponding laboratories in the international study (Tables 4 and 6).

**Table 5:** Intra-laboratory evaluation of best performing methods in human samples.

		N° Positive PCR/N° tested samples					
	ID	LbD2	LbD3	LbF1	LbQ	% pos	Cons
**Seropositive samples**	**4**	2/4	3/4	2/4	2/4	75	pos
	**6**	4/4	4/4	4/4	4/4	100	pos
	**11**	4/4	3/4	4/4	3/4	90,6	pos
	**15**	2/4	3/4	3/4	3/4	59,4	pos
	**32**	0/4	0/4	0/4	0/4	0	neg
**Seronegative samples**	**33**	0/4	0/4	0/4	0/4	0	neg
	**35**	0/4	0/4	0/4	0/4	0	neg
	**38**	0/4	0/4	0/4	0/4	0	neg
**Concordance**		28/32	29/32	29/32	28/32		

ID, sample identification number; LbX/1-6, Laboratory and test identification; % pos, Percentage of Positivity; Cons, Consensus PCR Result; pos, positive; neg, negative.

**Table 6:** Intra-Laboratory Evaluation of the four Best Performing Methods in samples from Table 5.

	PCR vs Consensus PCR of Table 5				PCR vs Serology			
Lb/Test	Se	Sp	Acc	kappa	Se	Sp	Acc	kappa
	N = 20	N = 12	N = 32	N = 32	N = 20	N = 12	N = 32	N = 32
**LbD2**	75	100	87,5	0.8	60	100	75	0.5
**LbD3**	81,25	100	90,6	0.8	65	100	78	0.6
**LbF1**	81,25	100	90,6	0.8	65	100	78	0.6
**LbQ**	75	100	87,5	0.8	60	100	75	0.5

LbX/1−6, Laboratory and test identification; Se, sensitivity; Sp, specificity; Acc, accuracy; kappa, kappa index.

## Discussion

PCR technology has been widely used for the diagnosis and monitoring of disease progression and therapy outcome in many infectious diseases [28]. Since 1989, PCR strategies have been developed aiming to analyse clinical samples infected with *T. cruzi* [7], [36]. However, each laboratory has applied its own protocols and quality controls, making comparison of PCR based findings among different research groups and geographical regions not reliable. This international collaborative study is a crucial first step aiming at the evaluation of currently used PCR procedures for detection of *T. cruzi* infection, towards the assessment of a standard operative procedure.

Out of the 48 PCR tests reported by 26 laboratories, those targeting ribosomal, miniexon or CO II subunit gene sequences were not sensitive enough when challenged against 10 fg/µl or less of purified DNA from the 3 tested parasite stocks, to merit further consideration. Thus, these methods appeared not suitable for sensitive molecular diagnosis of Chagas disease in clinical settings. However, these parasitic targets are been widely used for genotyping parasite discrete typing units [29]–[30], [37]–[41], mitochondrial gene haplotypes [42] or miniexon based *T. cruzi* I genotypes [43]–[44]. In this regard, multicentric evaluation and standardization of PCR based genotyping methods for identification of *T. cruzi* DTUs is needed.

### PCR Performance in Set A Samples

In set A, GPM included kDNA and sat-DNA PCR tests in similar proportions. However, Sat-DNA PCR tests were less sensitive than kDNA-PCR tests to detect *T. cruzi* I DNA. This is most likely due to the fact that *T. cruzi* DTU I harbors approximately four to ten-fold less number of satellite repeats than DTUs II, V and VI, which has been demonstrated by different molecular approaches [22], [45].

Regarding *T. cruzi* IV that also harbors a lower dosage for satellite sequences [22], similar analytical sensitivities of kDNA and Sat-DNA PCR tests were observed, being lower than that obtained for the other two tested DTU representative stocks. The genome size and relative DNA contents of Can III cl1 (116.44 Mb, 95% CI 110.4–122.63 and 1.090, respectively) and CL Brener (108.55 Mb, 95% CI 101.41–115.89 and 1.017) are similar [25], although Can III cl1 harbors about 5 fold less satellite repeats than CL-Brener [22]. The relative contribution of the nucleus and kinetoplast has not been measured but normally, kDNA represents 20–25% of the total DNA content [46]. There are no available data regarding the number of minicircles in the kinetoplast of Can III cl1, so it could be speculated that the lower analytical sensitivity of most PCR tests to detect DNA from this clone respect to the other ones, could be due to a lower minicircle copy dosage.

### PCR Performance in Set B Samples

Set B allowed evaluation of the influence of DNA extraction procedures in the PCR performance. A 72.2% of DNA extraction methods based on commercial kits led to GPM in set B, whereas 57.8% of phenol-chlorophorm extracted DNA led to GPM reports. These findings indicated that Guanidine Hidrochloride-EDTA blood was suitable for DNA extraction using kits based on lysis buffers containing Guanidine salts.

Out of the 25 GPM in set B, 14 had a sensitivity of 0.05 par/ml, which should be adequate for diagnosis of infection in chronic patients [20]. Indeed, the necessary detection limit in chronic Chagas disease has been stated as one parasite cell in 10 mL of blood [20].

### PCR Performance in Set C Samples

Analysis of PCR performance in set C clinical samples showed that the four best performing tests presented strong concordance with respect to consensus PCR results obtained by the 16 tests defined as GPM in sets A plus B (kappa index between 0.7 and 0.8). Out of them, three tests targeted sat-DNA sequences and only one targeted kDNA. These data are in agreement with previous works showing that PCRs targeting Sat-DNA performed better than PCRs targeting kDNA sequences [47]–[49], although kDNA based PCR has been more widely used [20].

Moreover, two of the sat-DNA best performing tests used Real Time PCR, one with a Sybr Green fluorescent dye (LbD2) and the other one with a TaqMan probe (LbF1). It must be pointed out that LbD2 and LbD3 tests were performed by the same laboratory. Out of the 16 GPM performed by 11 different laboratories, 3 laboratories performed two methods (LbD, LbK and LbP) and one lab developed 3 tests (LbG). These data point to laboratory dependence concerning PCR performance, which may be due to multiple factors including technical expertise, correct use of quality controls, instrumentation and reagents. For example, tests LbF1, LbS2 (GPM) and LbZ (not GPM) were all based on sat-DNA Real Time PCR using the same primer pair (cruzi 1 – cruzi 2), differing in the trade marks of the DNA extraction and Master Mix kits. Some tests shown as GPM in sets A+B had very low sensitivities in set C (LbK2, LbP2, LbV1, Table 4), suggesting that quality controls might have failed to distinguish false negative clinical samples.

A major drawback of most PCR tests is that they do not contain an internal amplification control (IAC). An IAC is a non target DNA sequence present in the same sample reaction tube, which is co-amplified simultaneously with the target sequence [50]. In a PCR without an IAC, a negative result can indicate that the reaction was inhibited, as a result of the presence of inhibitory substances in the sample matrix. The presence of PCR inhibition in Guanidine Hidrochloride-EDTA treated blood samples has been described [22]. The European Standardization Committee (CEN), in collaboration with International Standard Organization (ISO) has proposed a general guideline for PCR testing that requires the presence of IAC in the reaction mixture [51]. Therefore, only IAC-containing PCRs should undergo multicentre collaborative trials, which is a prerequisite for validation.

Some other tests shown as GPM in sets A+B had very low specificities (LbI2, LbW, LbG2, LbG3, Table 4). Amplicon carry-over contamination is one of the most probable causes. PCR master mixes with dUTP and Uracil-DNA N-glycosylase (UNG) intended to abolish amplicon carry-over contamination were used in some tests (LbF, LbG, LbL, LbS, Table 1). Nevertheless, some of them did not show good specificity in set C (LbG2, LbG3, LbG4, LbL1, LbL2, Table 4), suggesting that problems during sample processing, such as sample to sample contamination could have arisen. The median values of the sensitivities obtained in testing the Set C samples with the 16 tests determined to be GPMs by testing the Set A and Set B samples varied considerably depending on the clinical characteristics of the persons from whom the Set C samples were drawn. Indeed, sensitivity was 100% (25-75p = 100−100) for immunosuppressed heart transplanted pts, 56.5% (25-75p = 39.1–66.3%) for asymptomatic and 57.1% (25-75p = 14–75%) for symptomatic chronic Chagas disease patients. These data point to the limitations of PCR strategies for diagnosis of patients at the chronic phase of disease. In addition, some of these samples had been stored at 4°C for at least two years before this PCR study; thus higher PCR positivity might be obtained in prospective clinical studies but it is unlikely that the current PCR methods will have a sensitivity comparable to serological assays for diagnosis of chronic Chagas disease.

The four BPM methods were transferred to the Coordinating laboratory, where they were evaluated in a subset of clinical samples, each one tested in four independent assays, obtaining good concordance and confirming the performance reported by the participating laboratories in the previous international study (Tables 5 and 6). Further work is still needed to validate them through prospective studies in different settings. In this regard, this collaborative evaluation constitutes a starting point towards technical improvement and development of an international standard operating procedure (SOP) for *T.cruzi* PCR. In this context, the BPMs could be recommended for alternative diagnostic support, such as in the following settings: a) post-treatment follow-up of patients to look for failure of therapy to achieve parasitologic response [12], [14]–[16], [20], [22]; b) diagnosis of congenital Chagas disease in newborns in whom the presence of maternal anti-*T. cruzi* antibodies make serological studies useless [11], [15], [48]; c) early diagnosis of reactivation after organ transplantation of *T.cruzi* infected recipients under immunosuppressive therapy [18], [41], d) differential diagnosis of Chagas reactivation in patients with AIDS [39], and e) suspicion of oral transmission [52]. Moreover it can be useful for post-treatment follow-up of experimental animals to look for failure of therapy to achieve parasitologic cure [53]; in diagnosis in naturally infected triatomines or triatomines used for xenodiagnosis, since it has been shown that PCR tests are much more sensitive than microscopic examination of intestinal contents [37], [54]; and diagnosis of *T. cruzi* infection in mammalian reservoirs for which serologic tools have not been developed [38].
